# Apicortin, a Putative Apicomplexan-Specific Protein, Is Present in Deep-Branching Opisthokonts

**DOI:** 10.3390/biology14060620

**Published:** 2025-05-28

**Authors:** Ferenc Orosz

**Affiliations:** Institute of Molecular Life Sciences, HUN-REN Research Centre for Natural Sciences, Magyar Tudósok Körútja 2, 1117 Budapest, Hungary; orosz.ferenc@ttk.hu

**Keywords:** p25alpha domain, DCX domain, *Trichoplax adhaerens*, Apicomplexa, Choanoflagellata, Ctenophora, genome contamination

## Abstract

Apicortin, a tubulin/microtubule-binding protein, was first described in 2009 as a protein characteristic of apicomplexans; it was found to be present in all Apicomplexa genomes already sequenced. Apart from these, it was found only in *Trichoplax adhaerens*, the only known representative of Placozoa at the time. Subsequent analyses revealed that it is present in both closely and distantly related taxa of Apicomplexa (Chrompodellids, Squirmids, Dinoflagellates, and Perkinsids, i.e., in Myzozoa). On the other hand, it turned out that it is also present in early-branching fungi that reproduce by zoospores. Now, we have shown that apicortin is found in many deep-branching opisthokonts. In addition to these fungi and *T. adhaerens*, it is also present in other simple animals, including further Placozoa and Ctenophora, and another opisthokont clade, choanoflagellates. However, apicortin-homologous sequences detected in the genomes/transcriptomes of bilaterian animals are the result of contamination.

## Simple Summary

Apicortin is an enigmatic protein that was first described in 2009 as a protein characteristic of apicomplexan parasites. These unicellular organisms cause serious illnesses in humans and domestic animals. The most infamous apicomplexan species is *Plasmodium falciparum*, which causes malaria and kills over 1 million people each year. Other apicomplexans are responsible for numerous infectious diseases in wild and domesticated animals, such as coccidiosis, babesiosis, and toxoplasmosis, resulting in a significant economic burden for animal husbandry. In contrast to bacterial pathogens, these apicomplexan parasites share many metabolic pathways with their animal/human hosts. This makes drug development extremely difficult—a drug that harms an apicomplexan parasite is also likely to harm its human host. In this study, we show that apicortin also occurs in early-branching (very simple) animals and fungi, as well as in ‘Choanoflagellata’, which are the closest relatives of animals. This knowledge is important since it facilitates a better understanding of the features and functional roles of this protein, thus indirectly contributing to the fight against apicomplexan parasites.

## 1. Introduction

**Figure 1:**
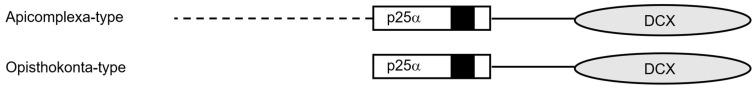
Schematic view of apicortins. The partial p25alpha and the DCX domains are indicated. The black squares label the position of the Rossmann-like motifs, and the dashed line corresponds to the disordered N-terminus of Apicomplexa-type apicortins.

Apicortin contains two tubulin/microtubule-binding domains that are connected by a linker of 30–40 amino acids. The N-terminal part contains the so-called partial p25alpha domain; the DCX (doublecortin) domain is in the C-terminal half [1]. In apicomplexan apicortins, the partial p25alpha domain is preceded by a long (70–80 aa) disordered region [2], as shown in Figure 1. The presence of the p25alpha domain (either the complete domain or its shorter forms) is characterized by a specific phylogenetic distribution: it is closely related to the existence of the eukaryotic flagellum/cilium [3,4]. In apicortin, only the C-terminal third of the complete p25alpha domain is present. Its amino acid sequence is quite conservative and usually contains the conserved motif GXGXGXXGR, often referred to as a ‘Rossmann-like motif’ [5]. The DCX domain is named after doublecortin, a brain-specific X-linked gene/protein [6]. The evolutionarily conserved DCX domain is about 80 amino acids long and usually occurs as a tandem repeat, mainly in animals [7]. It also occurs as a single domain in protists.

Apicortin was first described in 2009 as a protein characteristic of apicomplexans; it was found to be present in all Apicomplexa genomes sequenced up to that point [1]. Apart from these, it was found only in *Trichoplax adhaerens*, the only known representative of Placozoa at that time. Later, it turned out that it is present in both closely and distantly related taxa of Apicomplexa (Chrompodellids, Squirmids, Dinoflagellates, and Perkinsids, collectively termed Myzozoa) [8,9]. On the other hand, it became evident that it is also present in early-branching fungi, which reproduce by zoospores [4].

The presence of apicortin in Opisthokonta (*T. adhaerens*, flagellated fungi) raised the possibility that it can be found in further opisthokont species. Now, it will be shown that apicortin is found in other deep-branching opisthokonts. In addition to flagellated fungi and *T. adhaerens*, it is also present in other simple animals, including further placozoan species and Ctenophora, and in another opisthokont clade, choanoflagellates. However, apicortin-homologous sequences found in the genome/transcriptome of bilaterian animals are usually the result of contamination. Genome and transcriptome assembly data often contain DNA and RNA contaminations, originating from other organisms introduced during nucleotide sequencing, e.g., a large-scale search identified millions of contaminated entries in GenBank [10]. Similarly, various genomes and proteomes have been shown to be contaminated with genes/proteins of apicomplexan parasite origin as well [11,12,13].

## 2. Materials and Methods

The accession numbers of protein and nucleotide sequences refer to the National Center for Biotechnology Information (NCBI) GenBank database unless otherwise stated. NCBI Blast was used to search databases (http://www.ncbi.nlm.nih.gov/BLAST/ (accessed on 20 February 2025)) [14]. Whole sequences of various apicortins were used as queries against protein and nucleotide databases (including transcriptome shotgun assemblies [TSAs] and expressed sequenced tags [ESTs]) to find similar sequences in Opisthokonta using BLASTP and TBLASTN programs, respectively. The queries were *T. adhaerens* XP_002111209, *Babesia bovis* XP_001609847, *Plasmodium falciparum* XP_001351735, *Toxoplasma gondii* XP_002364910, *Jimgerdemannia flammicorona* RUS30044.1, and *Spizellomyces punctatus* XP_016606225.1. The hits were considered if the BLAST E-value was lower than 1 × 10^−5^; the query and the hit were reciprocal best hits [15], and the hit contained both the partial p25alpha and DCX domains. Then, the hits were used as queries in BLASTP and BLASTX searches to find the most similar protein for each. Genome GC ratios were retrieved from NCBI genome datasets (https://www.ncbi.nlm.nih.gov/datasets/genome/ (accessed on 8 May 2025)).

Multiple alignments of sequences were conducted by the Clustal Omega program (https://www.ebi.ac.uk/jdispatcher/msa/clustalo/ (accessed on 8 May 2025)) [16]. The N-terminal amino acids before the p25alpha domain were trimmed. Phylogenetic trees were constructed based on Bayesian and maximum-likelihood (ML) methods. Bayesian analysis, using MrBayes v3.1.2 [17], was performed to construct phylogenetic trees. The WAG model [18] with rate variation among sites, allowing for some sites to be evolutionarily invariable (WAG + G + I), and default priors were used. Two independent analyses were run with three heated and one cold chain (temperature parameter 0.2) for 1.6 × 10^−6^ generations, with a sampling frequency of 0.01, and the first 25% of generations were discarded as burn-in. The two runs were convergent, shown by the fact that the value of the potential scale reduction factor (PSRF) [19] approached 1. The MEGA11 program [20] was used for ML analysis. Bootstrap values were calculated by bootstrap analyses of 1000 replicates with the WAG + G + I model.

## 3. Results

### 3.1. Search for New Apicortins

Whole sequences of various apicortins (cf. Methods) were used as queries against protein and nucleotide NCBI databases to find similar sequences in Opisthokonta using BLASTP and TBLASTN analyses, respectively. The new hits were analyzed to determine whether they were real apicortins or the result of genome contamination.

The new apicortin-like sequences are listed in Table 1. Only one new (previously unknown) protein sequence was found, namely, *Hylaeus volcanicus* XP_053992704. *H. volcanicus* is an insect that undergoes complete metamorphosis and belongs to the Colletidae family. There were no new hits among the ESTs.

There were many more new hits among transcriptome (TSA) sequences (Table 1). Only hits in which both the p25alpha and DCX domains are present were considered. Most of the hits are sequences found in insect transcriptomes. Others belong to Amphibia, Crustacea, Placozoa, Ctenophora, and Choanoflagellata. Of course, a hit does not automatically mean that it is a real apicortin, as it may also indicate contamination of the given genome/transcriptome. This may especially be likely in species belonging to Bilateria.

The line in Table 1, ‘Placozoa-consensus’, refers to the sequence published in Ref. [21]. It is a consensus sequence based on several newly sequenced placozoan species, which have not yet been deposited in the NCBI database.

These hits were used as queries in BLASTP and BLASTX searches to find the most similar protein for each sequence (Table 2). In most cases, the most similar protein was found in Apicomplexa; in other cases, in Placozoa (*T. adhaerens*), in Chrompodellids (*Vitrella brassicaformis)*, and in Fungi (*Lobulomycetales* sp., *Blyttiomyces* sp.). The sequence identities, in general, are high (and the E-values are low); if not, it will be discussed later. Often, in the case of longer sequences, the TSA hit corresponds to more than one protein, and only one of them is an apicortin. This happens when the TSA is an ‘insect’ sequence, and the proteins are apicomplexan, most often *Gregarina niphandrodes* (as listed in Table 3). Some of these proteins have been found only in this species and do not possess any ortholog at all (XP_011128896, XP_011128900, XP_011128901, and XP_011128903).

### 3.2. Phylogenetic Analysis

**Figure 2:**
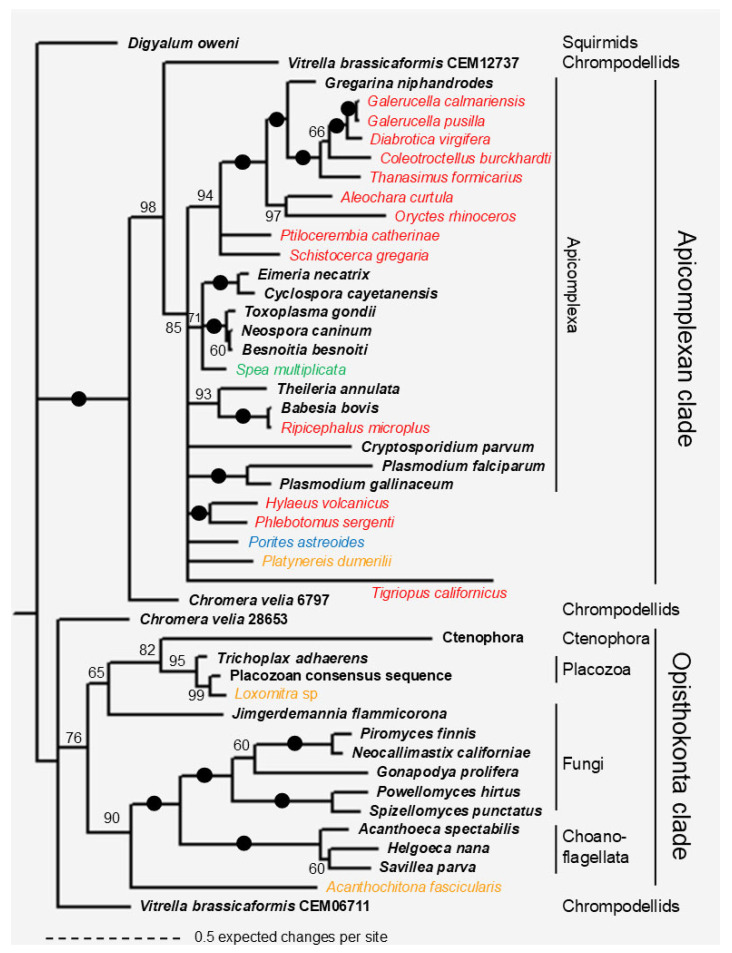
Phylogenetic tree of apicortin-like sequences by Bayesian analysis [17]. The root of the tree was chosen arbitrarily. Full circles at a node indicate that the branch was supported by maximal Bayesian posterior probability (BPP). All the other branches were supported by BPP, as indicated at the node. The accession numbers of proteins/TSAs are listed in Table S1. Operational taxonomic units marked with bold letters represent apicortins. (Tentative) contaminations found in Arthropoda (red), Spiralia (orange), Porifera (blue), and Amphibia (green) are labeled by colored letters.

**Figure 3:**
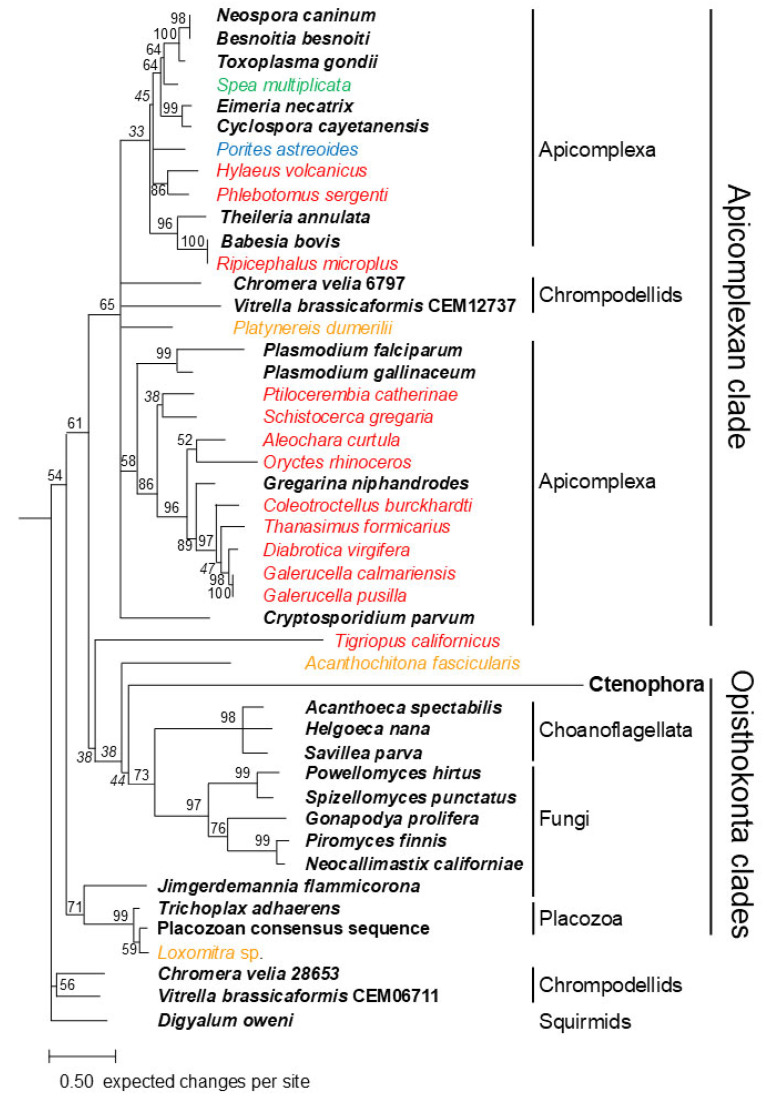
Phylogenetic tree of apicortin-like sequences by ML analysis [20]. The root of the tree was chosen arbitrarily. Numbers at a node indicate bootstrap values of 1000 replicates. The accession numbers of proteins/TSAs are listed in Table S1. Operational taxonomic units marked with uppercase letters represent apicortins. Operational taxonomic units marked with bold letters represent apicortins. (Tentative) contaminations found in Arthropoda (red), Spiralia (orange), Porifera (blue), and Amphibia (green) are labeled by colored letters.

Phylogenetic trees were constructed using Bayesian (Figure 2) and ML (Figure 3) methods. In addition to the newly identified apicortin-like sequences, apicomplexan (generally one per genus), chrompodellid (two per genus), a squirmid (Digyallum oweni), and fungal and placozoan apicortins were also included in the analysis. Some of the proteins contain a long N-terminus, which was not included in the alignment on which the analyses were based. Three apicortin-like sequences, previously shown to be present as contaminants in some species, were also included in the analyses. These are Aleochara curtula GATW02017439 [8,22], Porites astreoides GEHP01467367 [22], and Rhipicephalus microplus JT844686 [8].

The phylogenetic trees are quite similar; most importantly, there is a clade in both trees that contains all apicomplexan apicortins (“Apicomplexan clade”). The Bayesian tree reveals another clade containing fungal, placozoan, choanoflagellate, and ctenophora sequences (“Opisthokonta clade”). Within the latter clade, there are two sister groups: one of them contains apicortins of flagellated fungi and those of the Choanoflagellata, and the other one contains placozoan, ctenophora, and *Jimgerdamannia* apicortins. *J. flammicorona* is the only non-flagellated fungus that possesses an apicortin [4]. The ML tree also contains these two Opisthokonta groups, but they are not in a sister position.

One pair of chrompodellid apicortins (*Chromera velia* Cvel_6797 and *V. brassicaformis* CEM12737) is related to the apicomplexan clade; the other one differs significantly (*C. velia* Cvel_28653 and *V. brassicaformis* CEM06711), as can be seen from their position on the trees in accordance with data in the literature [8]. Bilaterian animal sequences are mostly located in the apicomplexan clade, except for *Loxomitra* sp. and *Acanthochitona fascicularis* sequences. The five insect-related apicortin-like sequences listed in Table 3, which are highly similar to *G. niphandrodes* apicortin, are sister to this Gregarinasina apicomplexan apicortin. Together, they are sisters to *Oryctes rhinoceros* and *A. curtula* sequences, which are also similar to *G. niphandrodes* apicortin (Table 1 and Ref. [8]).

### 3.3. Domains of Apicortin

**Figure 4:**
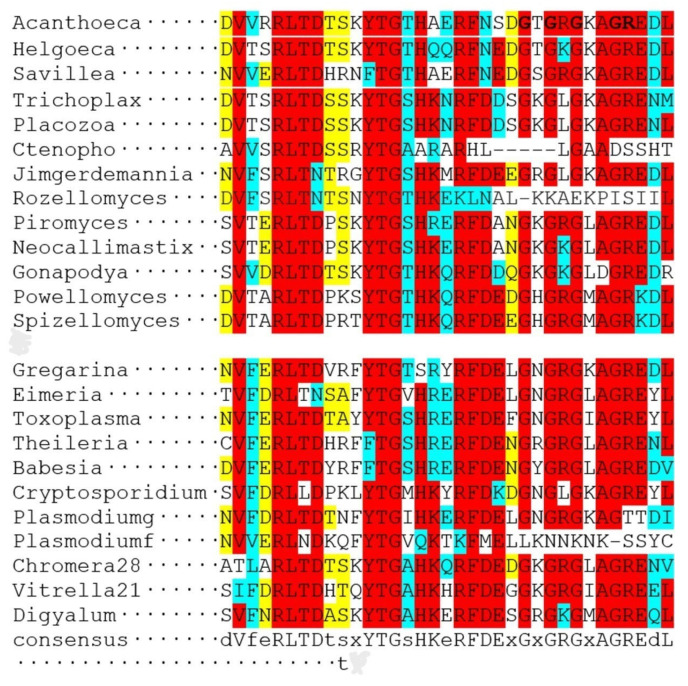
Multiple alignments of partial p25alpha domains of several apicortins by Clustal Omega [16]. In the upper and lower parts of the figure, there are opisthokont and myzozoan apicortins, respectively. Amino acids that are identical or biochemically similar in two-thirds of the proteins are marked with a red or blue background. A yellow background indicates that more than half of the amino acids are similar biochemically. The accession numbers of proteins/TSAs/SRAs are listed in Table S1.

**Figure 5:**
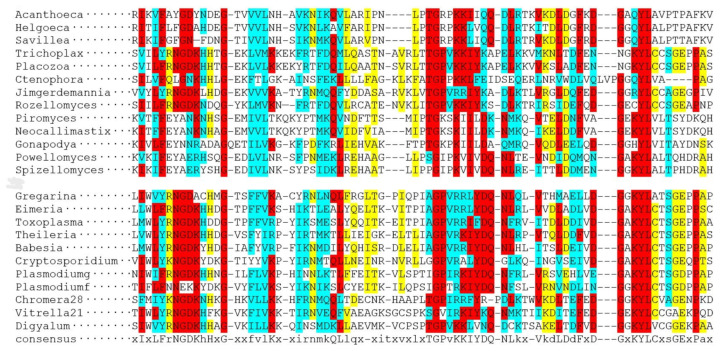
Multiple alignments of DCX domains of several apicortins by Clustal Omega [16]. In the upper and lower parts of the figure, there are opisthokont and myzozoan apicortins, respectively. Amino acids that are identical or biochemically similar in two-thirds of the proteins are marked with a red or blue background. A yellow background indicates that more than half of the amino acids are similar biochemically. The accession numbers of proteins/TSAs/SRAs are listed in Table S1.

Figure 4 and Figure 5 show the multiple alignments of the two characteristic domains of apicortins.

The partial p25alpha domain, in general, is the most conservative part of the molecule; the sequences are very similar, independent of whether the protein can be found in myzozoan or opisthokont species. However, there are three exceptions. *Rosella allomyces* (Fungi), *P. falciparum* (Apicomplexa), and *Ctenophora* sp. (Opisthokonta) lack the final part of this domain, which includes the Rossmann-like motif (Figure 4). The reason and the possible functional consequences are not known. Interestingly, in the *Plasmodium* genus, only species that parasitize mammals lack the Rossmann-like sequence. Species that occur in birds or reptiles (*Plasmodium gallinaceum, Plasmodium relictum)* do have this part of the sequence of the molecule (Figure 4). In the DCX domain, the overall similarity is somewhat lower between the two groups than in the case of the partial p25alpha domain; however, there is no exception: the similarity occurs through the whole domain in all orthologs (Figure 5).

## 4. Discussion

### 4.1. Phylogenetic Occurrence and Possible Function of Apicortins

Apicortin was found in 2009 as a characteristic protein of Apicomplexa [1]. This finding remains consistent: it is present in all Apicomplexa genomes [9], except in the smallest one, *Babesia microti* [23]. The namesake of Apicomplexa is the ‘apical complex’. One of the structural components of the apical complex is the conoid, which plays an important role in the invasion of the host cells by these parasites. It is likely that apicortin has some kind of role in providing the correct structure and function of the conoid. In *T. gondii*, apicortin was shown to be localized exclusively at the conoid and is essential for its structure and function [24,25]. A conoid consists of tubulin-based fibers. Thus, apicortin, which contains two tubulin-binding domains, p25alpha and DCX, is an ideal protein for its stabilization. *P. falciparum* does not have a conoid; here, apicortin is localized at the apical end of the parasites and is involved in the formation of the apical complex [26,27]. The observation that the drug tamoxifen hinders the growth of the asexual blood stage *P. falciparum*, which was attributed to the disruption of the apicortin–tubulin interaction observed in vitro, provides further evidence that apicortin has a critical function in apicomplexan microtubule function [26,27]. These data suggest that the presence of apicortin is correlated with that of the conoid and apical complex. This suggestion is corroborated by the fact that apicortin is present in other Myzozoa, namely, chrompodellids, squirmids, dinoflagellates, and perkinsids, which also possess a conoid or a conoid-like structure [28]. Moreover, it was demonstrated that the presence of apicortin is important in the parasite–host interaction; downregulation of apicortin leads to impaired host cell invasion [24,25,26].

Apart from these occurrences, apicortin was found originally in *T. adhaerens*, the only known representative of Placozoa at the time, where it is one of the most abundant proteins [29]. Placozoans belong to the simplest animals [30]; however, their exact phylogenetic position is debated [31,32,33,34]. Later, it became evident that in early-branching fungi, which reproduce by zoospores, apicortin is also present [4].

In the present work, it has been shown that not only *T. adhaerens* but also other recently discovered placozoans have apicortin, as well as another deep-branching animal, a ctenophore. *Trichoplax* sp. H2 [35] is a close relative of *T. adhaerens*; the apicortins of the two species are identical. A placozoan apicortin consensus sequence based on several newly sequenced placozoan species from the orders Hoilungea, Cladhexea, and Trichoplacea was also published [21] and used to produce a phylogenetic tree. This sequence shows 86% identity and 93% similarity with that of a *T. adhaerens* apicortin. Ctenophores, commonly known as comb jellies that inhabit marine waters worldwide, are one of the simplest non-bilaterian animals that are in strong “competition” with sponges for the title “the sister group to all other animals” [34,36,37]. The apicortin sequence of *Ctenophora* sp. shares a 31.25% identity and 47.22% similarity with that of the choanoflagellate *Helgoeca nana*. These values are about the same as those obtained in comparison with *Ctenophora* sp. and *T. adhaerens* apicortins (26.24% and 47.27%). This identity value is the lowest (and the E-value is the highest) in comparison with the other values discussed here. However, they are still significant enough to establish orthology between molecules of different phyla; *T. adhaerens* XP_002111209 and *Ctenophora* HBZJ01045600 are reciprocal best hits of each other. The ‘reciprocal best hit’ method helps to reveal a 1:1 orthology even in cases when the BLAST E-score is higher than 1 × 10^−10^ [15].

In addition to apicomplexans and ctenophores, Porifera (sponges) and Cnidaria are phyla of simple animals that do not belong to Bilateria. These phyla do not contain apicortin, but they do contain another p25alpha domain-containing protein, TPPP (tubulin polymerization promoting protein) [38]. On the one hand, it indicates that the correlation between the incidence of the p25alpha domain and that of the eukaryotic flagellum remains consistent; on the other hand, it suggests that apicortin and TPPP have similar functions, namely, stabilization of tubulin polymers. (An excellent review about TPPP, including its function in animals and humans, has been published recently [39].) In a cnidarian species, *Porites astreoides*, an apicortin-like sequence was found; however, it was shown to be a contamination of apicomplexan origin [22]. Its GC ratio (52.3%) is much higher than either the genome-wide GC ratio of *P. astreoides* (40%) [40] or that of the other animal (*T. adhaerens*) apicortin gene (39.7%).

Additionally, three species belonging to the Choanoflagellata [41], a sister group [42] to animals (Metazoa), also possess apicortin. (Choanoflagellata and Metazoa together form the Choanozoa clade [43].) All three Choanoflagellata belong to the Acanthoecidae family of the Acanthoecida order; apicortin appears to be absent in the other order of Choanoflagellates, Craspedida. In the species of this order, TPPP is present.

Of course, these opisthokont species do not have a conoid or apical complex. However, as mentioned, there is a strong correlation between the occurrence of the p25alpha domain and that of the eukaryotic flagellum/cilium, which are tubulin/microtubule-based structures. Apicomplexans also possess flagella that can only be found in male microgametes of these parasites; moreover, an evolutionary connection between the flagellum and apical complex was proposed [28,44]. Recent results suggest that the conoid complex evolved from flagellar components [45,46] or from the flagellar root apparatus [28,47]. Flagella and cilia are present in all animals; however, most fungi lack them, except the deeper branching clades that reproduce by motile zoospores propelled by a single, posteriorly oriented flagellum [48]. It was shown in several publications that there is a strict correlation between the incidence of the p25alpha domain and the flagellum in fungi, too [4,49,50]. As their name indicates, choanoflagellates also have a single flagellum.

The opisthokont apicortins lack the long, disordered N-terminal region [2,4], which was suggested to be necessary for the formation of the proper conoid structure in apicomplexans [25]. However, opisthokont apicortins do contain the two microtubule-binding domains (partial p25alpha and DCX); thus, it can be hypothesized that they play a role in the stabilization of flagellar microtubules. As long as this is just a hypothesis and we do not know the definitive answer, we also cannot answer the interesting question of why apicortin is lost in Bilateria.

### 4.2. Apicortin-like Contaminations in Animals

While the new choanoflagellate, placozoan, and ctenophora sequences are genuine apicortin hits, the other animal (Bilateria) hits appear to be from contamination. This is shown by their position on the phylogenetic trees. Most of them can be found within the apicomplexan clade, not within the Opisthokonta clade. If the homology between these sequences were due to real orthology, we would expect bilaterian species to be located in the Opisthokonta clade in the tree. If the bilaterian sequence is located within the Apicomplexa clade, then contamination or horizontal (lateral) gene transfer (HGT) may have occurred. HGT does not often happen from apicomplexans to animals (at least, the author has no information about it), although it is difficult to rule it out completely; however, there are examples in the opposite direction, from animals to Apicomplexa [51,52]. Bilaterian sequences that resemble the apicortin of *G*. *niphandrodes* (which is a Gregarinasina) (Table 2) are sisters to it and probably originate from (an) unknown yet non-sequenced Gregarinasina species. The high sequence identities (and low E-values) between such distant species also exclude the possibility of real orthology. Moreover, in several cases, the contamination is corroborated by a BLASTX search using the newly found TSAs as queries. In the case of longer sequences, the TSA hits correspond to more than one protein (two to ten), and only one of them is an apicortin. The other ones are various proteins, which are, in most cases, specific to Apicomplexa or even to a single apicomplexan species, such as *G. niphandrodes* (Table 3). The situation is the same in some other cases not shown in Table 3 (*Schistocerca gregaria* and *Ptilocerembia catheriane).* It is highly unlikely that 5–10 genes would have been transferred to animals at once by HGT; in these cases, it is much more likely that their presence was caused by contamination. Two further sequences in this ‘Gregarinasina’ clade are from *A. curtula* and *O. rhinoceros*, which are also similar to *G. niphandrodes* apicortin. *A. curtula* GATW02017439 was shown to be of Gregarinasina origin [8,22]; *O. rhinoceros* GHNO01082742 seems to also be a ‘foreign body’ in the insect transcriptome. This is suggested not only by its position in the tree and its sequence similarity to *G. niphandrodes* apicortin but also by its GC ratio of 43.1%, which is much higher than that of the whole genome (34.9%) [53] or the flanking TSA, GHNO01082741 (30.6%). The latter nucleotide is a real insect one, similar to TSAs in the same insect family, Scarabaeidae.

The *Rhipicephalus microplus* JT844686 sequence is sister to *B. bovis* apicortin, not surprisingly, since the sequence was shown to be of *B. bovis* origin [8]. In other cases, the source of the contamination is not so trivial. The *Spea multiplicate* TSA sequence, which is highly similar to *T. gondii* and *Neospora caninum* apicortins (Table 1 and Table 2), is within a clade on the tree that contains apicortins of the Eimeriorina suborder, to which these two apicortins belong.

On the Bayesian tree, there are only two bilaterian sequences in the Opisthokonta clade. Both of them are the most similar to *T. adhaerens* apicortin among the proteins of the NCBI database. *Loxomitra* sp. GIMU01103700 shows 85% identity and 91% similarity with *T. adhaerens* apicortin (Table S2). In comparison with the placozoan consensus apicortin sequence [21], these values are even higher: 89% identity and 95% similarity. These values are in the same range as between *T. adhaerens* and the placozoan consensus sequence (86% and 93%). Thus, it seems to be obvious that the sequence found in *Loxomitra* sp. originated from a placozoan species. This very high sequence identity suggests contamination, as HGT would have occurred some time ago, and the sequence may have changed significantly since then.

The habitat of placozoans and that of the species of the Loxosomatidae family are similar in the coastal zones of tropical and subtropical seas on various substrates such as rocks and corals [54]; thus, it can be imagined that the *Loxomitra* sample was indeed contaminated. The same phenomenon may be true for *A. fascicularis* (velvety mail shell) as well, which also lives in similar conditions; moreover, *Trichoplax* spp. are known to inhabit the shells of mollusks as well [55]. However, the identity/similarity between placozoan apicortins (*Trichoplax* spp., placozoan consensus sequence) and *A. fascicularis* is significantly lower (cca. 40/60%) than in the case of *Loxomitra* sp. It should be noted, however, that the *A. fascicularis* sequence is as similar to the chrompodellid *V. brassicaformis* apicortin (isolated from coral reefs) as it is to placozoan apicortins. However, it is interesting to compare the GC ratio of these apicortins/apicortin-like sequences: *T. adhaerens* 39.7%, *V. brassicaformis* 59.8%, and *A. fascicularis* 69.7%. This extremely high GC ratio is much higher than that of the whole genome of *A. fascicularis* (41%), suggesting the foreign origin of the apicortin-like sequence. Based on the similarity and GC ratio data, no definite statement can be made regarding the origin of the *A. fascicularis* sequence at this time. However, *T. adhaerens* cannot be the source of either contamination (cf. relatively low sequence similarity) or horizontal gene transfer (very different GC ratio).

Finally, the *Tigriopus californicus* sequence has different positions in the trees; in the Bayesian tree, it is located in the Apicomplexan clade, while in the ML tree, it is located outside the Apicomplexan clade in a sister position to the Opisthokonta clade. The *T. californicus* sequence shows a relatively low similarity to apicortin sequences (low identity percent, high E-value), so contamination is unlikely. It might be hypothesized that it is either a relic that has been uniquely preserved in this species, or rather its presence is the result of an ancient HGT. The latter case is made slightly more likely by the fact that the GC ratio of the apicortin-like sequence (39.0%) is slightly lower than the GC ratio of the whole genome of this species (42%).

In general, the presence of apicomplexan-derived contaminants in different genomes is not surprising. This has been previously pointed out by several authors [11,12,13]. In the case of wild animals, it is not possible to avoid infection by parasites before sequencing. Computational filters applied to the draft sequences are not always able to identify sequences of foreign origin. The taxonomic distribution of known complete genomes is not uniform. In the case of apicomplexan species, the genome/transcriptome is mainly known for species of medical/veterinary importance; however, for example, in the case of Gregarinasina, there are only a few fully sequenced species, although, as this study shows, they can often be sources of contamination. The knowledge of additional genomes will facilitate the bioinformatic filtering of contaminants and the identification of the source of contamination.

### 4.3. More General Evolutionary Considerations

**Figure 6:**
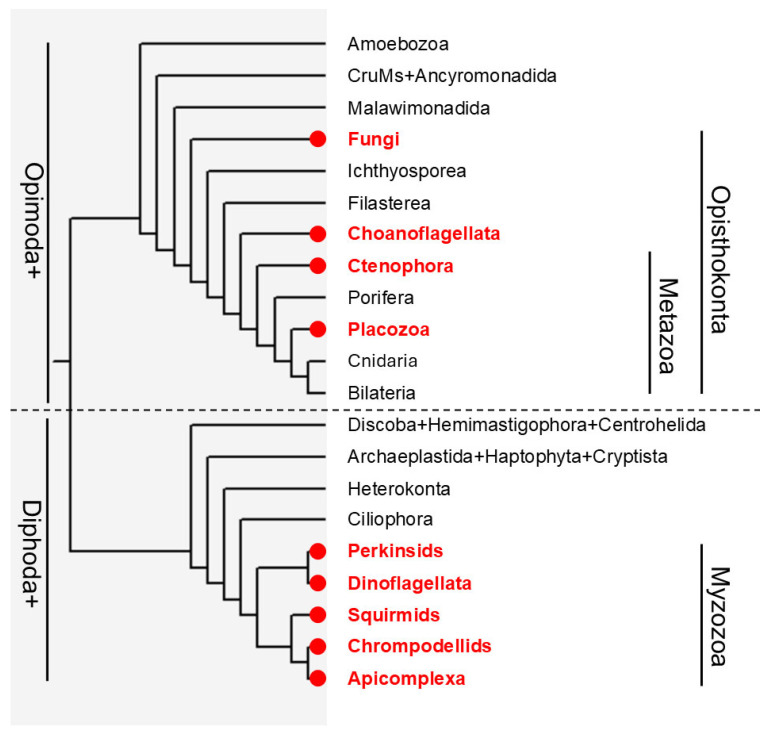
Occurrence of apicortin. The phylogenetic tree is based on Ref. [56]; the Myzozoan part is based on Ref. [57]. Red circles and the red color of operational taxonomic units indicate the presence of apicortin. Some lineages may not have sufficient data to definitively show absence.

For better visualization of the phylogenetic occurrence of apicortin, its presence/absence is shown on a eukaryotic evolutionary tree (Figure 6). It is based on earlier published trees [56,57] and shows the major lineages in eukaryotic supergroups, with some lineages lumped and others extended. Summarizing, it can be established that apicortin is present in two main clades. According to the more recent view, the two major eukaryotic groups are Opimoda+ and Diphoda+ [56,58]. Apicortin occurs in both ‘supergroups’. Its presence is limited to Myzozoa in Diphoda+ and to Opisthokonta in Opimoda.

Myzozoan apicortins were discussed in detail recently [9]. Now, it is just a reminder that apicortin was originally thought to be Apicomplexa-specific, but then it slowly became clear that it also occurs in other Myzozoa [9,10]. However, it is absent in Ciliophora, the sister group of Myzozoa [9]. This can be considered a fact, as several species belonging to Ciliophora have long had their whole genomes sequenced. Instead, they have another p25alpha domain-containing protein (usually several paralogs in a species), the so-called short-type TPPP [5].

Apicortin seems to be present in several early diverging lineages of Opisthokonta: early-branching Fungi, Choanoflagellata, and early-branching Metazoa. The Opisthokonta clade is composed of two main branches: Holomycota [59] and Holozoa [60]. The first contains, in addition to other groups, Fungi; the second contains, among others, Choanozoa [43]. The occurrence of apicortin in both main branches indicates that it was present in the Opisthokonta common ancestor. However, it seems that there were a few independent losses of this gene. Terrestrial fungi, which do not have zoospores, lost both flagella and apicortin. Some smaller Ophistokonta groups, such as Filasterea or Ichthyosporea, do not possess apicortin. Although as genome/transcriptome projects go on, this view may change, the lack of apicortin in these groups is in accordance with the fact that Filasterea and Ichthyosporea also lost their flagellum [41]. In Metazoa, Porifera and the (Cnidaria + Bilateria) clade also lack apicortin; instead, they contain another p25alpha domain-containing protein, TPPP. It means two further losses in Porifera and in the common ancestor of Cnidaria and Bilateria. It is worth noting that Figure 6 represents the most accepted view that Placozoa is sister to (Cnidaria + Bilateria); however, there is also support for the case that (Placozoa + Cnidaria) is sister to Bilateria [37,61]. If Placozoa forms a clade with Cnidaria, it would imply another gene loss in the ancestor of Cnidaria.

This phylogenetic distribution suggests that apicortin was either present in the last eukaryotic common ancestor and lost in many lineages or it underwent a horizontal transfer involving an Apicomplexa/Myzozoa to an ancestral opisthokont.

## 5. Conclusions

Apicortin was thought to be specific to the phylum Apicomplexa [1]. This statement is still valid; it is present in almost all apicomplexan genomes [9]. However, it has become obvious that its incidence is significantly broader. On the one hand, it can be found in Myzozoa (i.e., in Alveolata phyla, except Ciliata); on the other hand, it is present in simple Opisthokonta as well. The latter includes early-branching flagellated Fungi [4], Choanoflagellate, the sister clade to Metazoa (animals), and simple animals, such as Placozoa [1] and Ctenophora. However, it is absent in bilaterian animals. Apicortin-homologous sequences in the genome/transcriptome of these animals are the result of contamination. In some cases, its source can be exactly identified; in other cases, only the genus, family, or order can be suggested.

## Figures and Tables

**Table 1:** List of new apicortin-like sequences.

Species	Accession Number ^1^	Taxonomy	E-Value ^2^ *T. adhaerens*	E-Value ^3^ *T. gondii*
*Hylaeus volcanicus*	XP_053992704	Insecta/Endopterygota	6 × 10^−33^	4 × 10^−90^
*Schistocerca gregaria*	GJPN010108154	Insecta/Polyneoptera	4 × 10^−33^	4 × 10^−77^
*Phlebotomus sergenti*	GKTC01023996	Insecta/Endopterygota	6 × 10^−26^	9 × 10^−91^
*Galerucella calmariensis*	HAMF01019916	Insecta/Endopterygota	3 × 10^−20^	2 × 10^−62^
*Galerucella pusilla*	HAMG01049297	Insecta/Endopterygota	3 × 10^−20^	2 × 10^−62^
*Diabrotica virgifera*	GHNJ01033740	Insecta/Endopterygota	4 × 10^−16^	1 × 10^−52^
*Oryctes rhinoceros*	GHNO01082742	Insecta/Endopterygota	2 × 10^−14^	2 × 10^−48^
*Ptilocerembia catherinae*	GDBY01042306	Insecta/Polyneoptera	2 × 10^−13^	6 × 10^−74^
*Coleotroctellus burckhardti*	GJXT01027847	Insecta/Paraneoptera	4 × 10^−13^	6 × 10^−46^
*Thanasimus formicarius*	GDPC01032790	Insecta/Endopterygota	8 × 10^−11^	1 × 10^−42^
*Tigriopus californicus*	GHUE01002433	Arthropoda/Crustacea	3 × 10^−7^	2 × 10^−19^
*Diaphanosoma celebensis*	GGQP01033578	Arthropoda/Crustacea	5 × 10^−13^	4 × 10^−7^
*Loxomitra* sp. *KK-2020*	GIMU01103700	Spiralia/Entoprocta	9 × 10^−104^	8 × 10^−34^
*Acanthochitona fascicularis*	GJAX01016147	Spiralia/Mollusca	3 × 10^−25^	2 × 10^−23^
*Platynereis dumerilii*	HBZZ01068313	Spiralia/Annelida	4 × 10^−24^	8 × 10^−79^
*Spea multiplicata*	GKIA01122747	Chordata/Amphibia	3 × 10^−28^	9 × 10^−115^
*Trichoplax* sp. H2 TR738	GFSF01001671	Placozoa	2 × 10^−119^	2 × 10^−31^
Placozoa	Consensus ^4^	Placozoa	3 × 10^−113^	4 × 10^−36^
*Ctenophora* environmental sample	HBZJ01045600	Ctenophora	2 × 10^−5^	-
*Acanthoeca spectabilis*	GGPA01011677	Choanoflagellata	4 × 10^−18^	2 × 10^−17^
*Helgoeca nana*	GGOR01004317	Choanoflagellata	3 × 10^−18^	2 × 10^−17^
*Savillea parva*	GGOL01031575	Choanoflagellata	5 × 10^−13^	8 × 10^−15^

^1^ The accession numbers refer to TSAs, except XP_053992704, which is a protein. ^2^ E-values were obtained in searches where *T. adhaerens* XP_002111209 was used as the query. ^3^ E-values were obtained in searches where *T. gondii* XP_002364910 was used as the query. ^4^ From Ref. [21].

**Table 2:** Proteins that are the most similar to novel apicortin-like sequences.

Species	Accession Number ^1^	Most Similar Protein		E-Value ^4^	Percent Identity ^4^
		Species ^2^	Accession Number ^3^		
*Hylaeus volcanicus*	XP_053992704	*Neospora caninum*	XP_003883150	2 × 10^−87^	65.57%
*Schistocerca gregaria*	GJPN010108154	*Besnoitia besnoiti*	XP_029217437	2 × 10^−47^	50.50%
*Phlebotomus sergenti*	GKTC01023996	*Neospora caninum*	XP_003883150	3 × 10^−84^	63.55%
*Galerucella calmariensis*	HAMF01019916	*Gregarina niphandrodes*	XP_011128898	2 × 10^−77^	73.26%
*Galerucella pusilla*	HAMG01049297	*Gregarina niphandrodes*	XP_011128898	3 × 10^−77^	73.26%
*Diabrotica virgifera*	GHNJ01033740	*Gregarina niphandrodes*	XP_011128898	3 × 10^−68^	71.15%
*Oryctes rhinoceros*	GHNO01082742	*Gregarina niphandrodes*	XP_011128898	6 × 10^−60^	59.54%
*Ptilocerembia catherinae*	GDBY01042306	*Porospora* cf. *gigantea B*	XP_068375468	1 × 10^−51^	61.98%
*Coleotroctellus burckhardti*	GJXT01027847	*Gregarina niphandrodes*	XP_011128898	2 × 10^−62^	67.36%
*Thanasimus formicarius*	GDPC01032790	*Gregarina niphandrodes*	XP_011128898	6 × 10^−56^	70.68%
*Tigriopus californicus*	GHUE01002433	*Plasmodium gallinaceum*	XP_028530755	3 × 10^−10^	35.03%
*Diaphanosoma celebensis*	GGQP01033578	*Vitrella brassicaformis*	CEL97699	1 × 10^−11^	34.59%
*Loxomitra* sp.	GIMU01103700	*Trichoplax adhaerens*	XP_002111209	7 × 10^−102^	84.66%
*Acanthochitona fascicularis*	GJAX01016147	*Trichoplax adhaerens*	XP_002111209	9 × 10^−24^	38.69%
*Platynereis dumerilii*	HBZZ01068313	*Sarcocystis calchasi*	CAL7857217	6 × 10^−57^	70.48%
*Spea multiplicata*	GKIA01122747	*Neospora caninum*	XP_003883150	2 × 10^−100^	71.43%
*Trichoplax* sp. H2	GFSF01001671	*Trichoplax adhaerens*	XP_002111209	2 × 10^−119^	100%
Placozoa	consensus	*Trichoplax adhaerens*	XP_002111209	3 × 10^−113^	85.80%
*Ctenophora*	HBZJ01045600	*Trichoplax adhaerens*	XP_002111209	6 × 10^−3^	26.24%
*Acanthoeca spectabilis*	GGPA01011677	*Lobulomycetales* sp.	KAL3897002	5 × 10^−24^	43.31%
*Helgoeca nana*	GGOR01004317	*Lobulomycetales* sp.	KAL3897002	3 × 10^−26^	42.24%
*Savillea parva*	GGOL01031575	*Blyttiomyces* sp.	KAJ3329948.1	4 × 10^−21^	38.24%

^1^ The accession numbers refer to TSAs, except XP_053992704, which is a protein. ^2^ Color code: no color: Apicomplexa; yellow: Chrompodellids; blue: Placozoa; green: Fungi. ^3^ The accession numbers refer to proteins. ^4^ E-values and identity percentages were obtained in searches where hits listed in the second column were used as queries.

**Table 3:** *Gregarina niphandrodes* proteins corresponding to TSA sequences found in insects.

TSA	*Galerucella calmariensis* HAMF01019916	*Galerucella pusilla* HAMG01049297	*Thanasimus fornicarius* GDPC01032790	*Diabrotica virgifera* GHNJ01033740	*Coleotroctellus burckhardti* GJXT01027847
*Gregarina niphandrodes* proteins and E-values	XP_011128895 1.2 × 10^−100^	XP_011128895 2 × 10^−100^			
	XP_011128896 5.7 × 10^−7^	XP_011128896 2.8 × 10^−7^			
	XP_011128897 1.6 × 10^−33^	XP_011128897 5.8 × 10^−33^			
	XP_011128899 1 × 10^−49^	XP_011128899 1.5 × 10^−49^			
	**XP_011128898** 1.9 × 10^−82^	**XP_011128898** 2.7 × 10^−82^	**XP_011128898** 4.9 × 10^−59^	**XP_011128898** 3 × 10^−73^	**XP_011128898** 2 × 10^−67^
	XP_011128900 7.5 × 10^−16^	XP_011128900 1.1 × 10^−15^	XP_011128900 3.8 × 10^−17^	XP_011128900 8 × 10^−15^	XP_011128900 5 × 10^−5^
	XP_011128901 1.6 × 10^−68^	XP_011128901 2.4 × 10^−68^	XP_011128901 8.6 × 10^−74^	XP_011128901 2 × 10^−72^	
	XP_011128902 1.1 × 10^−147^	XP_011128902 9.2 × 10^−147^	XP_011128902 6.8 × 10^−127^	XP_011128902 4 × 10^−152^	
		XP_011128903 0	XP_011128903 0		
		XP_011128904 1 × 10^−64^	XP_011128904 2.4 × 10^−137^		
			XP_011128905 0		

A yellow background indicates proteins specific to *G. niphandrodes.* A lilac background indicates very small (≤9 × 10^−100^) E-values. The accession number of an apicortin is bold.

## Data Availability

The data presented in this study are available in this paper and in the Supplementary Material.

## Supplementary Materials

The following supporting information can be downloaded at: https://www.mdpi.com/article/10.3390/biology14060620/s1, Table S1: Accession numbers of proteins/TSAs/SRAs shown in Figure 2, Figure 3, Figure 4 and Figure 5 [62]. Table S2: Pairwise identity/similarity between three apicortin-(like) sequences. Figure S1: Multiple alignments of apicortins. Figure S2: Bayesian tree for Figure 2. Figure S3: ML tree for Figure 3.

## Abbreviations

The following abbreviations are used in this manuscript:

BLAST	Basic Local Alignment Search Tool
BPP	Bayesian Posterior Probability
EST	Expressed Sequenced Tag
DCX	Doublecortin
HGT	Horizontal Gene Transfer
ML	Maximum Likelihood
NCBI	National Center for Biotechnology Information
PSRF	Potential Scale Reduction Factor
SRA	Sequence Read Archive
TSA	Transcriptome Shotgun Assembly
TPPP	Tubulin Polymerization Promoting Protein
