# Supplementary material for: Apicortin, a Putative Apicomplexan-Specific Protein, Is Present in Deep-Branching Opisthokonts
Source: Biology (Basel). 2025 May 28;14(6):620. doi: 10.3390/biology14060620 (PMC12189498; doi:10.3390/biology14060620)
Supplement: Supplementary file 1 [file biology-14-00620-s001.zip › biology-3594513-supplementary.pdf]

Table S1. Accession Numbers of proteins/TSAs/SRAs shown in Figures 2-5

Name on Figures 4, 5	Species	NCBI Accession Number		
		Protein	TSA	Sequence Read Archive (SRA)
Trichoplax	*Trichoplax adhaerens*	XP_002111209		
Placozoan cons. seq.	Placozoan consensus seq.	OG14524*		
Ctenophora	Ctenophora enviromental sample		HBZJ01045600	SRR1296727, SRR1296843
Acanthoeca	*Acanthoeca spectabilis*		GGPA01011677	SRR6344971
Helgoeca	*Helgoeca nana*		GGOR01004317	SRR6344981
Savillea	*Savillea parva*		GGOL01031575	SRR6344983
Jimgerdemannia	*Jimgerdemannia flammicorona*	RUS30044		
Piromyces	*Piromyces finnis*	ORX59328		
Neocallimastix	*Neocallimastix californiae*	ORY36261		
Gonapodya	*Gonapodya prolifera*	KXS19308		
Powellomyces	*Powellomyces hirtus*	KAI8919344		
Spizellomyces	*Spizellomyces punctatus*	XP_016606225		
Rozella	*Rozella allomycis*	RKP19964		
Chromera 28653	*Chromera velia*	Cvel_28653**		
Chromera 6797	*Chromera velia*	Cvel_6797**		
Vitrella (CEM)6711	*Vitrella brassicaformis*	CEM06711		
Vitrella(CEM)12737	*Vitrella brassicaformis*	CEM12737		
Digyalum	*Digyalum oweni*		GHRU01063100	SRR9831920, SRR9831921
Gregarina	*Gregarina niphandrodes*	XP_011128898		
Eimeria	*Eimeria necatrix*	XP_013434470		
Cyclospora	*Cyclospora cayetanensis*	XP_026194446		
Toxoplasma	*Toxoplasma gondii*	XP_002364910		
Besnoitia	*Besnoitia besnoiti*	XP_029217437		
Neospora	*Neospora caninum*	XP_003883150		
Theileria	*Theileria annulata*	XP_952938		
Babesia	*Babesia bovis*	XP_001609847		
Cryptosporidium	*Cryptosporidium parvum*	XP_001388280		
Plasmodiumf	*Plasmodium falciparum*	XP_002808695		

Plasmodiumg	*Plasmodium gallinaceum*	XP_028530755		
Porites	*Porites astreoides*		GEHP01467367	SRR2046818 – SRR2046825
Rhipicephalus	*Rhipicephalus microplus*		JT844686	-
Aleochara	*Aleochara curtula*		GATW02017439	SRR921563
Hylaeus	*Hylaeus volcanicus*	XP_053992704		
Schistocerca	*Schistocerca gregaria*		GJPN010108154	SRR15423963 – SRR15423967
Phlebotomus	*Phlebotomus sergenti*		GKTC01023996	SRR27378302, SRR27378310, SRR27378311
Galerucellac	*Galerucella calmariensis*		HAMF01019916	-
Galerucellap	*Galerucella pusilla*		HAMG01049297	-
Diabrotica	*Diabrotica virgifera*		GHNJ01033740	SRR7818012 – SRR7818015
Oryctes	*Oryctes rhinoceros*		GHNO01082742	SRR9208137 – SRR9208140
Ptilocerembia	*Ptilocerembia catherinae*		GDBY01042306	SRR1811989
Coleotroctellus	*Coleotroctellus burckhardti*		GJXT01027847	SRR18778725
Thanasimus	*Thanasimus formicarius*		GDPC01032790	SRR2083732
Tigriopus	*Tigriopus californicus*		GHUE01002433	SRR8167998
Loxomitra	*Loxomitra* sp. *KK-2020*		GIMU01103700	SRR11344291
Acanthochitona	*Acanthochitona fascicularis*		GJAX01016147	SRR13862571 – SRR13862584
Platynereis	*Platynereis dumerilii*		HBZZ01068313	ERR11027265, ERR11027269, ERR11027292, ERR11027295, ERR11027297, ERR11027299, ERR11028157, ERR11028161
Spea	*Spea multiplicata*		GKIA01122747	SRR16201678 – SRR16201691

*Ref. [21]. https://www.frontiersin.org/articles/10.3389/fevo.2022. 1016357/full#supplementary-material: Supplementary Data 2. The full sequence is: MSKKSQDVTSRLTDSSKYTGSHKNRFDDSGKGLGKAGRENLVEYTGSTSSQSRDFGVTKGNVEKSDKPVVSGGLGKEKFGVQADKPISVILFRNGDK HHTGEKLVLKKEKYRTFDQILQAATNSVRLTTGPVKKIYKAPELKKVVKSLADFENNGKYLCCSGEPPASKDKLPAALF. **CryptoDB (Ref. [62]).

Table S2. Pairwise identity/similarity between three apicortin-(like) sequences

	Trichoplax adhaerens	Placozoa consensus sequence	*Loxomitra* sp.
Trichoplax adhaerens	100%	86/93%	85/91%
Placozoa consensus sequence	86/93%	100%	89/95%
Loxomitra sp.	85/91%	89/95%	100%

Figure S1: Multiple alignment of apicortins

```
#NEXUS
[TITLE: Written by EMBOSS 27/11/24]

begin data;
dimensions ntax=46 nchar=180;
format interleave datatype=protein missing=X gap=-;

matrix
Digyalum             MSVFNRLTDASKYTGAHKERFDESGRGKGMAGREQLVNYTGDTQSSNRDA
Chromera28           DATLARLTDTSKYTGAHKQRFDEDGKGRGLAGRENVTHHDGSTESAVRSH
Ctenophora           TAVVSRLTDSSRYTGAARARHL-----LGAADSSH--TSRGGVESKTLSE
Trichoplax           QDVTSRLTDSSKYTGSHKNRFDDSGKGLGKAGRENMVDYTGSTSSQSRDF
Vitrella15_brassicaf RNIFEKLCDPSLYTGAHRERFDENGKGRGLAGREYVYYVDGMTESPTRCH
Placozoa             QDVTSRLTDSSKYTGSHKNRFDDSGKGLGKAGRENLVEYTGSTSSQSRDF
Jimgerdemannia1      SNVFSRLTNTRGYTGSHKMRFDEEGRGLGKAGREDLVEFDGLTPSPSRSK
Piromyces            QSVTERLTDPSKYTGSHRERFDANGKGRGLAGREDLCINDGNTSSKSRNH
Neocallimastix       QSVTERLTDPSKYTGSHKERFDANGKGKGLAGREDLCINDGNTSSASRNH
Gonapodya            PSVVDRLTDTSKYTGTHKQRFDDQGKGKGLDGREDRVNFDGNTQSESRSN
Powellomyces         ADVTARLTDPKSYTGTHKQRFDEDGHGRGMAGRKDLVDYDGSTTSAHREH
Spizellomyces1       ADVTARLTDPRTYTGTHKQRFDEEGHGRGMAGRKDLVEYDGNTTSAHRGH
Vitrella21_brassicaf QSIFDRLTDHTQYTGAHKHRFDEGGKGRGIAGREELVNIDGSTESSARRH
Chromera67           PSVWERLTDPSRYTGVHRERFDEFGRGRGLAGRENVYYFDGMTESPSRCH
Gregarina            RNVFERLTDVRFYTGTSRYRFDELGNGRGKAGREDLNVKTGWAPCPQRPT
Eimeria              KTVFDRLTNSAFYTGVHRERFDELGNGRGLAGREYLYAHDGMTESPSRTH
Cyclospora           KTVFDRLTDSAFYTGAHRERFDELGNGRGLAGREYLYTHDGMTESPSRTH
Toxoplasma           KNVFERLTDTAYYTGSHRERFDEFGNGRGIAGREYLYAYDGLTESPSRCH
Neospora             KNVFERLTDTAYYTGSHRERFDEFGNGRGIAGREYLYAYDGLTESPSRCH
Besnoitia            KNVFERLTDTAYYTGSHRERFDEFGNGRGIAGREYLYAYDGLTESPSRCH
Theileria            RCVFERLTDHRFFTGSHRERFDENGRGRGLAGRENLYFFDGNTESYSRVH
Babesia              RDVFERLTDYRFFTGSHRERFDENGYGRGLAGREDVYIFDGNTESVSRPH
Cryptosporidium      TSVFDRLLDPKLYTGMHKYRFDKDGNGLGKAGREYLFREDGYTESTKRKH
Plasmodiumf          TNVFERLNDKQFYTGVQKTKFMELLKNNKN-----------KSSYCYNNI
Plasmodiumg          KNVFDRLTDTNFYTGIHKERFDELGNGRGKAGTTDIYIHDGWTQAKTRNH
Hylaeus              RNVWERLTDYQLYTGIHKERFDEFGNGRGLAGREYVYLHDGMTESPSRTH
Phlebotomus          RNVWDRLTDHQYYTGSHKERFDEFGNGRGLAGREYVFLHDGMTESPMRCH
Spea                 SNVFDRLTDYTYYTGVHRERFDEFGNGRGLAGREYLYAYDGMTESPSRCH
Porites              RSVFDRLTDPTYYTGTHRERFDEVGNGRGLAGREYLCVHDGLTESPSRTH
Rhipicephalus        RDVFERLTDYRFFTGSHRERFDENGYGRGLAGREDVYIFDGNTESVSRPH
Ptilocerembia        RNVFDRLTDHRFYTGIHRERFDEFGNGRGKAGREYVYLASGWTPAYTRPN
Aleochara            RSVFERLSDHRFYTGTARFRFDELGNGRGKAGREDLHIDSGWAPSPARPS
Galerucellac         LSVFERLTDVRYYTGTSRFRFDELGNGRGKAGREDMYLGTGWAPTASRPT
Galerucellap         LSVFERLTDVRYYTGTSRFRFDELGNGRGKAGREDMYLGTGWAPTASRPT
Oryctes              RNVFSRLSDPRYYTGSSRQRFDELGNGRGLAGRETLILDTGWTKSCRHQQ
Platynereis          RQVFDRLTDPQYYTGAHKERFDEFGVGRGLAGREYIYLNDGWTESAARSH
Schistocerca         RDVFSRLTDHRFYTGIHRQRFDEFGNGRGRAGREYMFVADGWTHCESRPK
Diabrotica           XXXXXXXXXXXXXXXXXRFRFDELGNGRGKAGREDMYLKTGWAPTQCRPT
Loxomitra            QDVTSRLTDSSKYTGSHKNRFDGSGKGLGKAGRENLVDYTGSTSSQSRDF
Acanthochitona       SDVFDRLSDPKAFTGAHKERFDEEGKGRGKAGRVEAEDETKDLSQMTRPG
Tigriopus            RNVFDRLTNVDNYPQAHRRRFDKYGRGRGLLGTEERAVVHKHGS-IVWPE
Thanasimus           XXXXXXXXXXXXXXXXXXXXXXELGNGRGKAGREDLYLKTGWAPCAARPN
Coleotroctellus      XXXXXXXXXXXXXXXXXXXXXXXXXXXXXKAGRENMYLNTGWPATTDRPN
Acanthoeca           PDVVRRLTDTSKYTGTHAERFNSDGTGRGKAGREDLIAYDGSTNATQRKT
Helgoeca             DDVTSRLTDTSKYTGTHQQRFNEDGTGKGKAGREDLMIDDGSTNAARKA-
Savillea             PNVVERLTDHRNFTGTHAERFNEDGSGRGKAGREDLIAYDGSTNAAERKA

Digyalum             NVTDTV---GN--------RQHKDVVKGSMGQQKFGTQAETPISIWVYRN
Chromera28           AIEKTVDE--K--------AHKKSVVKGPLGQQKFGTQADTPISFMIYKN
Ctenophora           VVARTG---YK-------KPQDKTQSA------RFAAST---VSILVFQL
Trichoplax           AVNKSN---VS--------KSDKPVVASALGKEKFGTQMDKPISVILYRN
Vitrella15_brassicaf EVYSSVRE-RP--------KRKPLRRPPALKERMSG-APPRAKLIWLYRN
Placozoa             GVTKGN---VE--------KSDKPVVSGGLGKEKFGVQADKPISVILFRN
Jimgerdemannia1      APRHTAALNPS--------VSKTPVVKGPLGTQKFGTQAR---VVYLYRN
Piromyces            TIDNS----------VEPRPKSKPVVH---KVDNYGV---TAKKVTFFEY
Neocallimastix       TIENS----------VEPRPKNKPVVH---KVNNYGT---TAKKITFFEY
Gonapodya            LKKSQELRKSQELGKSNDSLSKKPVVK---PKDDFGV---KPKKIVLFEY
Powellomyces         RPYGSDMDLNARH------DHEKPIVRSRSRETDIGV---TAKKVKIFEY
Spizellomyces1       VPFGSDQDLRERV------DREKPIVRSKSRESDIGV---TAKKIKIFEY
```

```
Vitrella21_brassicaf AVEKTVDHVER--------AGRKPVVQGALGQQKFGTQAETPITIWLYRN
Chromera67           EIYSTVVT-QK---------RKAAVTPGTLGVQKFGTQAVTPKLIWVYRN
Gregarina            EVYSSALR-VM---------RQPVVEPGTLGVQKFGTQAIPPKLIWVYRN
Eimeria              EVYSSVIR-RP---------RRATVTPGTLGVQRFGLQTSPPRLLWLFRN
Cyclospora           EVYSSVVR-RP---------RKALVAPGTLGVQRFGLQTSPPRLLWLFRN
Toxoplasma           EVYSSVIK-RP---------RKPVVTPGTLGIQRFGVQIPAPRLMWLYRN
Neospora             EVYSSVVK-RP---------RKPVVTPGTLGVQRFGVQIPAPRLMWLYRN
Besnoitia            EVYSSVVK-RP---------RKPVVTPGTLGVQRFGVQIPAPRLMWLYRN
Theileria            EVYSSVLP-RQ---------RRPMVPPNTLGVKKFGVQIEPPKLVWLYRN
Babesia              EVYSTVLR-GP---------PKTVVPRGVLGLQKFGVQIATPKLMWLYRN
Cryptosporidium      EVVSSSIK-RH--------SYANISS---------NDFLQKAKVIWLYKN
Plasmodiumf          KVFSTMLK-KP---------CNYVVTPGTLGIQKYGIQTGRPKTIFLFNN
Plasmodiumg          EIYSSDIK-KS--------KKNPVVTPGTLGIQKYGIQIASPKNIWIFRN
Hylaeus              EVFSSSIR-YP---------RQPVVEPGMLGIQRFGVQTITPKLMWLYRN
Phlebotomus          EVFSTIVK-QP---------RKPVVEPGTLGIQRYGVQTITPKLMWLYRN
Spea                 EVYSTVIK-RP---------RRPVVTPGTLGVQRFGVQVAPPKLMWLYRN
Porites              EVFSSVAK-RP---------RKPVIPAGTLGVQRYGTQVATPPLMWLFRN
Rhipicephalus        EVYSTVLR-GP---------PKTVVPRGVLGLQKFGVQIATPKLMWLYRN
Ptilocerembia        EIYSSVIK-KP---------RKPVVEPGTLGVQKFGVQTITPKLIWVFRN
Aleochara            QVYSSAWK-FP---------REPVVEPGTLGVQLFGVQTITPKLIWVYSN
Galerucellac         EVYASILR-AP---------RNPVIEPGTLGVQKFGTQAIPLKMIWLYRN
Galerucellap         EVYASILR-AP---------RNPVIEPGTLGVQKFGTQAIPLKMIWLYRN
Oryctes              EVYSTNLK-AP---------RPYVVEPGTLGVQKFGVQTITPKTVWIYRN
Platynereis          EVYSSVVR-KP---------RRAVVTPGTLGIQKFGVQAVTPRLLWLYRN
Schistocerca         EVYSTCIK-KA---------RRPVVEPGTLGVQKYGVQVSTPRLIWVYRN
Diabrotica           EVYASILR-PA---------RNPVIEPGTLGVQKFGTQAIPLKMIWLYRN
Loxomitra            GVNKGN---AE--------KSDKPVVSGGLGKEKFGVQTDKPINVYFFRN
Acanthochitona       VAKGSDRR-AS---------GAAERPKRASAVEKFGVQADKAANAYFFKN
Tigriopus            TVYVV--------------KDNNLPLPCTIGLQKFGIQILQPPKIYIHSV
Thanasimus           NVYASALR-LP---------RNPVIEPGTLGVQKFGTQAIALKMIWLFRN
Coleotroctellus      EVYASVLR-LP---------RHPVIEPGTLGVQKFGTQAIPLKLIWIYRN
Acanthoeca           IKSQG---------------KGSRVVVKKWDGQKFGTQADMPRRIKVFAY
Helgoeca             PSPTA---------------RTSKVVVKKWDGKKFGTQADMPRRITIFLF
Savillea             VKSTGA--------------QHSKVSVHKWDGKVFGTQADVPRRIKIFGF

Digyalum             GDKHHAGVKILLKK-QINSMDKLLAEVMK-VCPSPTGPVKKLVNQ-----
Chromera28           GDKNHKGHKVLLKK-HFRNMQQLTDECNK-HAAPLTGPIRRFYR-P----
Ctenophora           GNKHHLGEKFTLGKA-INSFEKLLLLFAG-KLKFATGPPKKLFEIDSEQE
Trichoplax           GDKHHTGEKLVMKKEKFRTFDQMLQASTN-AVRLTTGPVKKIYKAP----
Vitrella15_brassicaf GDKKHLGSPSFVRP-HVQSLDLLYREASA-EIRLLTGPVKRLYDQ-----
Placozoa             GDKHHTGEKLVLKKEKYRTFDQILQAATN-SVRLTTGPVKKIYKAP----
Jimgerdemannia1      GDKLHDGEKVVVKA-TYRNMQQFYDDASA-RVKLVTGPVRRIYKA-----
Piromyces            ANKNHSGEMIVLTKQKYPTMKQVNDFTTS---MIPTGKSKIILDK-----
Neocallimastix       ANKNHAGEMVVLTKQKYPTMKQVIDFVIA---MIPTGKSKIILDK-----
Gonapodya            NNRADAGQETILVKGKFPDFKRLIEHVAK---FTPTGKPKIILDA-----
Powellomyces         AERHSQGEDLVLNR-SFPNMEKLREHAAG---LLPSGIPKVIVDQ-----
Spizellomyces1       AERYSHGEDIVLNK-SYPSIDKLREHAAT---LIPPGIPKVIVDQ-----
Vitrella21_brassicaf GDKHFKGVKFIVKK-TIRNVEQFVAEAGKSGCSPKSGVIRKIYKQ-----
Chromera67           GDRSHEGHPVYLRN-SIKTMEFLYRECTK-VACPFTGPVLKIYDQ-----
Gregarina            GDACHMGTSFFVKA-CYRNLNQLFRGLTG-PIQPIAGPVRRLYDQ-----
Eimeria              GDKHHDGTPFFVKS-HIKTLEALYQELTK-VITPIAGPVRRLYDQ-----
Cyclospora           GDKHHDGTPFFVKA-HIKTLEALYQELTK-VVTPIAGPVRRLYDQ-----
Toxoplasma           GDKHDDGTPFFVRP-YIKSMESLYQQITK-EITPIAGPVRRIFDQ-----
Neospora             GDKHDDGTPFFVRP-YIKTMESLYQQITK-EITPIAGPVRRIFDQ-----
Besnoitia            GDKHDDGTPFFVRP-YIKTMESLYQQITK-EITPIAGPVRRIFDQ-----
Theileria            GDKHHDGVSFYIRP-YIRTMKTLLIEIGK-ELTLIAGPVRKIYDQ-----
Babesia              GDKYHDGIAFYVRP-FIKNMDILYQHISR-DLELIAGPVRRIYDQ-----
Cryptosporidium      GDKYDKGTIYYVKP-YIRNMTQLLNEINR-NVRLLGGPVRALYDQ-----
Plasmodiumf          EKKYDKGVYFLVKS-YIKNIKSLCYEITK-ILQPSIGPTRKIYDQ-----
Plasmodiumg          GDKHHNGILFLVKP-HINNLKTLFFEITK-VLSPTIGPIRKIYDQ-----
Hylaeus              GDKHHAGSPYFLKS-HVKTMQFFYQEATK-VVEPIAGPVRKIYDQ-----
Phlebotomus          GDKNFDGSPYFVKR-HIKTLEALYQDATK-LVKPVAGPVRRLYDQ-----
Spea                 GDKHHDGTPFFLRS-HIKSMQAFYQEITK-IITPIAGPLRRIFdQ-----
Porites              GDKHHDGIPFFLRP-HIKTLELLYQEASR-KVTPIAGPVRLIYDQ-----
Rhipicephalus        GDKYHDGIAFYVRP-FIKNMDILYQHISR-DLELIAGPVRRIYDQ-----
Ptilocerembia        GDELHHGEAIFLRK-HISSMEHLHNELTK-VLKPYGGPARIIYDQ-----
Aleochara            GNEFDLGTSFLVKS-YYKRIPQLHRALSD-KIRLYAGPCRRLYDQ-----
Galerucellac         GDACHLGENFFVKA-VYKDLTQLLRGISQ-QVRPIGGPARRLYDQ-----
Galerucellap         GDACHLGENFFVKA-VYKDLTQLLRGISQ-QVRPIGGPARRLYDQ-----
```

```
Oryctes              GNPDDTGKSVLVKN-CYKNLQVFIRNLP---IQLVAGQPRRLYDQ-----
Platynereis          NDGHHSGTTYFLRS-HVRNLDTLYAECTK-MLQLPTGPVRRLYDQ-----
Schistocerca         GDMCDEGTPIFVKN-HIRNLEQLHRLLTQ-EVKPFGGPSRRLYDQ-----
Diabrotica           GDACHKGENFFVKS-VYKDLTQLLRGVSG-QLKPIGGPARRLYDQ-----
Loxomitra            GDKHHAGEKVVMKKEKFRTFDQILQAATN-SVRLTTGPVKKIYKAP----
Acanthochitona       GDKHDKGTKVVVGL-NFNTWEQLLKRLSK-VLGLPTGPVKKVLHVRDGPH
Tigriopus            YD--NKIQSIVIRN-HIKKWSVLQNEIFK-FFPDVKSPYVYLWDM-----
Thanasimus           GDACHKGKNFFVKS-SYRDLSQLLRGITE-QLHPIAGPARRLYDQ-----
Coleotroctellus      GDASHKGESLFIKT-CYKDLNQLLRGVTQ-QCAPIAGPARRMYDQ-----
Acanthoeca           GDY-NDEGTVVVLNHAVKNIKQVLARIPN----LPTGRPKKIIQQ-----
Helgoeca             GDA-HDEGTVVVLNHSVKNLKAVFARIPN----LPTGRPKKLVQQ-----
Savillea             GN--FDNGTIVVLNHSIKNMKQVLARVPN----LPTGRPKKLIQQ-----

Digyalum             --DCKTSAKELTDFVDGGKYLVCGGEKPAA
Chromera28           --DLKTWVKDLTEFEDGGKYLCVAGENPKD
Ctenophora           --RLNRVW-DLVQLVPGGQYLVA----PAG
Trichoplax           --ELKKVMKNLTDFENNGKYLCCSGEPPAS
Vitrella15_brassicaf --HL-EAIRDIDDLTDGGKYLCCAGEQPCM
Placozoa             --ELKKVVKSLADFENNGKYLCCSGEPPAS
Jimgerdemannia1      --DLKTLVRGLDQFEDGGRYLCCAGEGPIV
Piromyces            --NM-KQVTELDNFVAGEKYLVLTSYDKQH
Neocallimastix       --NM-KQIKELDDFVAGEKYLVLTSYDKQH
Gonapodya            --QM-RQVQDLEELQDGGHYLVITAYDNSK
Powellomyces         --NL-TEVNDIDQMQNGAKYLALTQHDRAH
Spizellomyces1       --NL-REITTLDDMENGGKYLALTPHDRAH
Vitrella21_brassicaf --NMKTIIKDITEFEDGEKYLCCGAEKPQD
Chromera67           --NL-KRVKKLEHFVDGGKYLCCGGELPSL
Gregarina            --NL-QLVTHMAELLDGGKYLATSGEPPAP
Eimeria              --NL-RLVVDLADLVDGAKYLCTSGEPPSC
Cyclospora           --NL-RIIGDLADLVDGAKYLCTSGEPPAC
Toxoplasma           --NF-RVITDLDDIVDGAKYLCTSGEPPAA
Neospora             --NF-RVVTDLEDIVDGAKYLCTSGEPPAA
Besnoitia            --NF-RVVTDLEDIVDGAKYLCTSGEPPAA
Theileria            --NL-RPVTQLDDFVDGAKYLCTSGEPPAS
Babesia              --NL-HLITSLDEIVDGAKYLCTSGEPPAP
Cryptosporidium      --GL-KQINGVSEIVDGAKYLCTSGEQPTS
Plasmodiumf          --NF-SLVRNVNDLINGGKYLCTSGDPPAP
Plasmodiumg          --NF-RLVRSVEHLVEGAKYLCTSGDPPAP
Hylaeus              --NL-NVVSRLEDIIEGGKYLCSSGELPAN
Phlebotomus          --QL-RPVQYLEDLVDGGKYLCTSGEPPTF
Spea                 --nl-qvvvdledvvDGAKYLCTSGEPPAS
Porites              --NL-RPLRSLHEIVDGGKYLCTSGEPPAR
Rhipicephalus        --NL-HLITSLDEIVDGAKYLCTSGEPPAP
Ptilocerembia        --NL-ERINHIAELVDGAKYLCTSGEPPAC
Aleochara            --NM-KLVTHVAELVDGAKYLATSGEPPAP
Galerucellac         --NL-QLVTELPELVDGAKYLVTSGEPPAP
Galerucellap         --NL-QLVTELPELVDGAKYLVTSGEPPAP
Oryctes              --NL-KEITHLAELVDGAKYLVTAGDRPVE
Platynereis          --NL-QPVRQLSDIVDGAKYLCCSGEPPAR
Schistocerca         --HF-QPVRHLCEIIDGAKYLCTSGEPPAP
Diabrotica           --NL-QLITELPELVDGAKYLVTSGEPPAP
Loxomitra            --ELKKALKNLTDFENNGKYLCCSGELPAS
Acanthochitona       GNRH-VRVRDLDDILDGECYLCCGAEPLSR
Tigriopus            --NL-RRVLTVNDFVHGAKYLMTTTKKVNA
Thanasimus           --NL-ILVTDFTHLVDGAKYLVTSALSLTS
Coleotroctellus      --NL-QLITHITQLVDGAKYLITSGEPPAV
Acanthoeca           --DLRTKVKDLDGFKDGAQYLAVPTPAFKV
Helgoeca             --DLRTKITDLDGFRDGGQYLALPTPAFKV
Savillea             --DLRTRVKDLDGFRDGGQYLALPTTAFKV
;

end;
begin assumptions;
options deftype=unord;
end;
```

Figure S2: Bayesian tree for Fig. 2.

```
#NEXUS

[ID: 8484712715]
begin trees;
   [Note: This tree contains information on the topology,
          branch lengths (if present), and the probability
          of the partition indicated by the branch.]
   tree con_50_majrule =
(Digyalum:0.301666,((Vitrella15_brassicaf:0.435788,((((Gregarina:0.099645,((((Ga
lerucellac:0.004958,Galerucellap:0.005422)1.00:0.038090,Diabrotica:0.050684)1.00
:0.068026,Coleotroctellus:0.158206)0.66:0.035455,Thanasimus:0.152921)1.00:0.1215
06)1.00:0.079906,(Aleochara:0.171790,Oryctes:0.374598)0.97:0.073926)1.00:0.17933
5,Ptilocerembia:0.187233,Schistocerca:0.220939)0.94:0.124197,((Eimeria:0.031055,
Cyclospora:0.056599)1.00:0.138392,(Toxoplasma:0.029225,(Neospora:0.005286,Besnoi
tia:0.005336)0.60:0.009842)1.00:0.093331,Spea:0.086418)0.71:0.054654,(Theileria:
0.173485,(Babesia:0.005737,Rhipicephalus:0.005318)1.00:0.185490)0.93:0.120290,Cr
yptosporidium:0.620342,(Plasmodiumf:0.480596,Plasmodiumg:0.089685)1.00:0.224023,
(Hylaeus:0.180127,Phlebotomus:0.138729)1.00:0.081310,Porites:0.188632,Platynerei
s:0.246306,Tigriopus:1.167078)0.85:0.093079)0.98:0.131317,Chromera67:0.180727)1.
00:0.356946,(Chromera28:0.268865,(((Ctenophora:1.035541,(Trichoplax:0.033216,(Pl
acozoa:0.038558,Loxomitra:0.059685)0.99:0.048407)0.95:0.138180)0.82:0.201334,Jim
gerdemannia1:0.436736)0.65:0.078513,(((((Piromyces:0.067839,Neocallimastix:0.033
979)1.00:0.300888,Gonapodya:0.428192)0.60:0.083788,(Powellomyces:0.130979,Spizel
lomyces1:0.099604)1.00:0.384508)1.00:0.199546,(Acanthoeca:0.100972,(Helgoeca:0.1
82742,Savillea:0.154691)0.60:0.034114)1.00:0.537198)1.00:0.191345,Acanthochitona
:0.709527)0.90:0.165005)0.76:0.117734,Vitrella21_brassicaf:0.278764)0.61:0.07844
0);

   [Note: This tree contains information only on the topology
          and branch lengths (mean of the posterior probability density).]
   tree con_50_majrule =
(Digyalum:0.301666,((Vitrella15_brassicaf:0.435788,((((Gregarina:0.099645,((((Ga
lerucellac:0.004958,Galerucellap:0.005422):0.038090,Diabrotica:0.050684):0.06802
6,Coleotroctellus:0.158206):0.035455,Thanasimus:0.152921):0.121506):0.079906,(Al
eochara:0.171790,Oryctes:0.374598):0.073926):0.179335,Ptilocerembia:0.187233,Sch
istocerca:0.220939):0.124197,((Eimeria:0.031055,Cyclospora:0.056599):0.138392,(T
oxoplasma:0.029225,(Neospora:0.005286,Besnoitia:0.005336):0.009842):0.093331,Spe
a:0.086418):0.054654,(Theileria:0.173485,(Babesia:0.005737,Rhipicephalus:0.00531
8):0.185490):0.120290,Cryptosporidium:0.620342,(Plasmodiumf:0.480596,Plasmodiumg
:0.089685):0.224023,(Hylaeus:0.180127,Phlebotomus:0.138729):0.081310,Porites:0.1
88632,Platynereis:0.246306,Tigriopus:1.167078):0.093079):0.131317,Chromera67:0.1
80727):0.356946,(Chromera28:0.268865,(((Ctenophora:1.035541,(Trichoplax:0.033216
,(Placozoa:0.038558,Loxomitra:0.059685):0.048407):0.138180):0.201334,Jimgerdeman
nia1:0.436736):0.078513,(((((Piromyces:0.067839,Neocallimastix:0.033979):0.30088
8,Gonapodya:0.428192):0.083788,(Powellomyces:0.130979,Spizellomyces1:0.099604):0
.384508):0.199546,(Acanthoeca:0.100972,(Helgoeca:0.182742,Savillea:0.154691):0.0
34114):0.537198):0.191345,Acanthochitona:0.709527):0.165005):0.117734,Vitrella21
_brassicaf:0.278764):0.078440);
end;
```

Figure S3: ML tree for Fig. 3.

```
(((((((((Neospora,Besnoitia):0.00084474,Toxoplasma):0.11802925,Spea):0.01262596
,(Eimeria,Cyclospora):0.16373094):0.06676806,Porites,(Hylaeus,Phlebotomus):0.132
31929):0.01517931,(Theileria,(Babesia,Rhipicephalus):0.23017376):0.20993481):0.0
6576056,Chromera67,Vitrella15_brassicaformis,Platynereis,((Plasmodiumf,Plasmodiu
mg):0.32221423,((Ptilocerembia,Schistocerca):0.02356493,((Aleochara,Oryctes):0.0
8017896,(Gregarina,(Coleotroctellus,(Thanasimus,(Diabrotica,(Galerucellac,Galeru
cellap):0.03524397):0.06942036):0.03848561):0.14148075):0.07969007):0.22131940):
0.17869677):0.03811525,Cryptosporidium):0.25092796,(Tigriopus,(Acanthochitona,(C
tenophora,((Acanthoeca,Helgoeca,Savillea):0.69626238,((Powellomyces,Spizellomyce
s1):0.37719623,(Gonapodya,(Piromyces,Neocallimastix):0.39932528):0.14031673):0.4
2222551):0.22003409):0.04142457):0.25836178):0.00003174):0.17069507,(Jimgerdeman
nia1,(Trichoplax,(Placozoa,Loxomitra):0.04865239):0.38523490):0.13583186):0.1213
6920,(Chromera28,Vitrella21_brassicaformis):0.03290217,Digyalum:0.42948861);
```

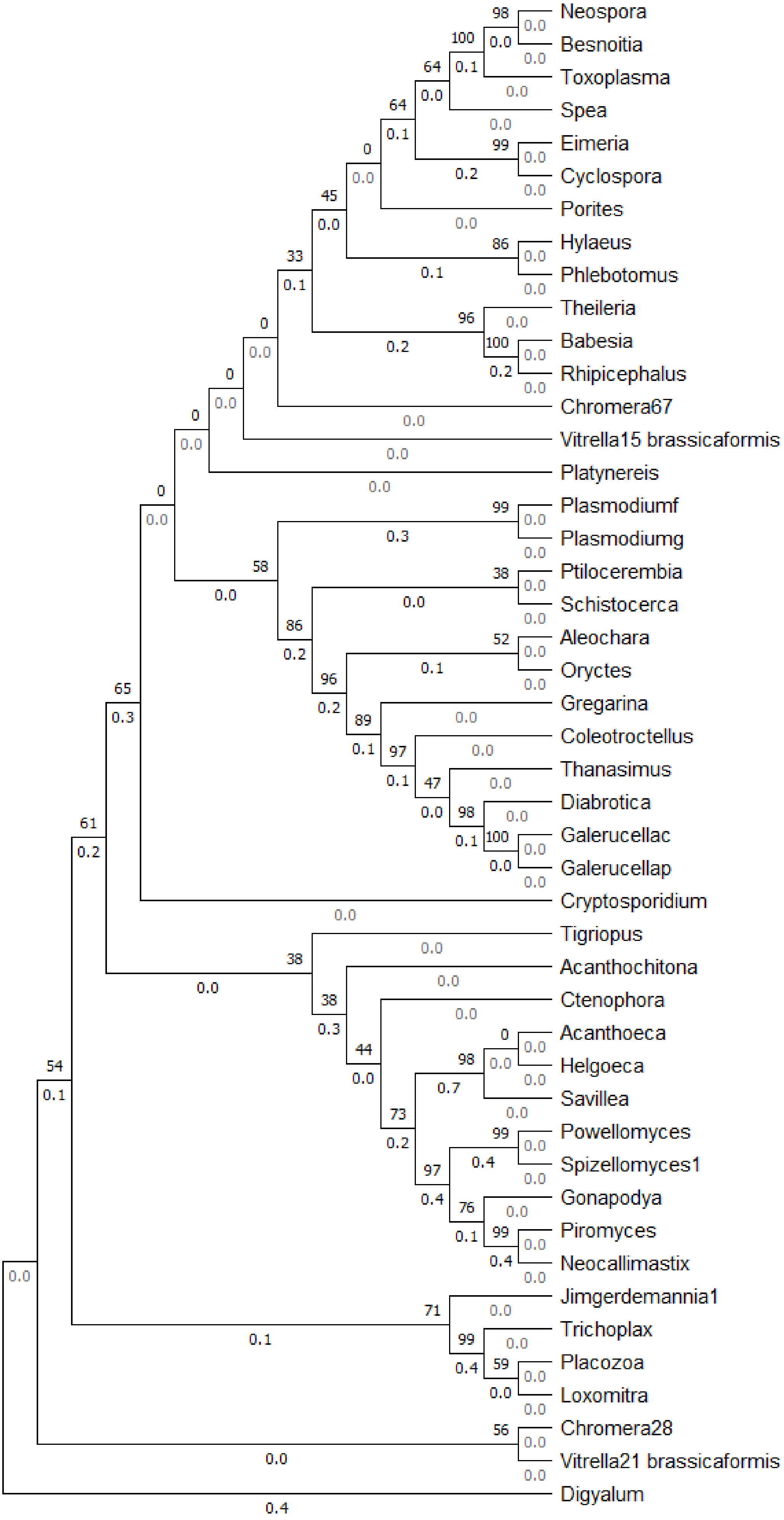
Neospora
Besnoitia
Toxoplasma
Spea
Eimeria
Cyclospora
Porites
Hylaeus
Phlebotomus
Theileria
Babesia
Rhipicephalus
Chromera67
Vitrella15 brassicaformis
Platynereis
Plasmodiumf
Plasmodiumg
Ptilocerembia
Schistocerca
Aleochara
Oryctes
Gregarina
Coleotroctellus
Thanasimus
Diabrotica
Galerucellac
Galerucellap
Cryptosporidium
Tigriopus
Acanthochitona
Ctenophora
Acanthoeca
Helgoeca
Savillea
Powellomyces
Spizellomyces1
Gonapodya
Piromyces
Neocallimastix
Jimgerdemannia1
Trichoplax
Placozoa
Loxomitra
Chromera28
Vitrella21 brassicaformis
Digyalum
